# A new Education Section for *FEBS Open Bio*

**DOI:** 10.1002/2211-5463.12214

**Published:** 2017-04-03

**Authors:** Angel Herráez, Luciane V. Mello

**Affiliations:** ^1^ Department of Systems BiologyUniversity of AlcaláAlcalá de HenaresSpain; ^2^ School of Life SciencesUniversity of LiverpoolUK

Following a proposal initiated by the FEBS Education Committee, *FEBS Open Bio* is starting a section devoted to original and valuable publications in biochemical and molecular life sciences education. We have been entrusted with the task of coordinating this new Section.

The overall aim of this initiative is, in the spirit of FEBS and its Education Committee, to foster good educational practices, stimulate the development of innovative teaching methods, and disseminate advice on educational techniques and resources.

We are seeking to receive contributions in the form of research articles dealing with educational issues, with a clear structure of hypothesis, methods, and validation of results. Among the topics of manuscripts we encourage are those dealing with best practice, innovative methods, teaching bioinformatics, and use of technology in education. In addition, articles more specifically aimed at internationalization, training or career planning will also be welcome. The main focus, in line with the mission of FEBS, is on biochemistry and other molecular life sciences, but the scope of the section widens to include molecular and cellular sciences in general.

The FEBS Education Committee recognizes that there are other educational materials – such as practical papers on teaching methods, reports on education experiences, reviews of educational resources – that will not fit the format or requirements of a journal paper. Plans are underway to provide an online forum for discussion and sharing of such materials later in 2017. This initiative will complement the Education Section in *FEBS Open Bio*.

Authors wishing to contribute to the Education Section of the journal should submit their articles to https://mc.manuscriptcentral.com/febsopen. These articles will be sent out for peer review, as for other regular submissions to the journal. However, authors of accepted articles will be exempt from the article publication fee, and all publication costs will be covered by FEBS. Articles will be published in the monthly issues of the journal and will also be available as a Virtual Issue on Education.

We look forward to receiving your contributions.

